# Efficient Inverted Perovskite Solar Cells Utilizing Inorganic Composite Multiple Electron Transport Layers

**DOI:** 10.1002/smll.202411978

**Published:** 2025-07-25

**Authors:** Annan Zhu, Hao Gu, Wang Li, Jia Guo, Shengwen Li, Gang Wang, Junmin Xia, Chao Liang, Shi Chen, Guichuan Xing

**Affiliations:** ^1^ Joint Key Laboratory of the Ministry of Education Institute of Applied Physics and Materials Engineering University of Macau Avenida da Universidade Taipa Macau 999078 P. R. China; ^2^ State Key Laboratory of Organic Electronics and Information Displays Nanjing University of Posts and Telecommunications Nanjing 210000 P. R. China; ^3^ MOE Key Laboratory for None‐quilibrium Synthesis and Modulation of Condensed Matter School of Physics National Innovation Platform (Center) for Industry‐Education Integration of Energy Storage Technology Xi'an Jiaotong University Xi'an 710000 P. R. China

**Keywords:** inorganic electron transport layer, perovskite solar cells, thermal evaporation, tin oxide, tungsten‐doped zinc oxide

## Abstract

Electron transport layers (ETLs) featuring optimal film coverage and favorable electronic properties play a critical role in high‐performance perovskite solar cells (PSCs). In contrast to organic ETLs, which have high material costs, inorganic metal oxide ETLs are considered promising alternatives for efficient inverted PSCs because of their low cost, high carrier mobility, and excellent stability. However, fabricating high‐quality top inorganic ETLs that preserve the active perovskite layer remains a challenge. Herein, a composite electron transport bilayer comprising atomically coherent interfaced tin dioxide (SnO_2_) nanoparticles and tungsten‐doped zinc oxide (WZO) is introduced, which further facilitates charge extraction and mitigates detrimental interfacial deprotonation reactions. The tungsten doping ratio can be precisely controlled by adjusting the co‐evaporation parameters. The results reveal that tungsten enhances charge extraction by fine‐tuning the energy levels, whereas the SnO_2_ layer simultaneously passivates the perovskite/ETL interface defects and inhibits deprotonation reactions. Utilizing this inorganic composite multiple architecture, a record efficiency of 23.19% is achieved for inverted PSCs with an all‐inorganic ETL. This cost‐effective approach provides a viable pathway for industrial‐scale production of high‐performance PSCs.

## Graphical abstract

In this article, a composite layer comprising SnO_2_ nanoparticles and tungsten‐doped zinc oxide (WZO) is used as an inorganic electron transport layer in inverted perovskite solar cells (PSCs). WZO balances energy levels, and SnO_2_ passivates interface defects and inhibits deprotonation reactions. This cheap approach provides a viable pathway for the industrial‐scale production of high‐performance PSCs.

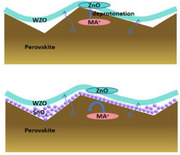

## 1 Introduction

Perovskite solar cells (PSCs) have demonstrated remarkable potential for addressing growing global energy demands.^[^ ^1^ ^]^ As an efficient and cost‐effective photovoltaic technology, a steady increase in the power conversion efficiency (PCE) of PSCs has been achieved, exceeding 26.7%, which is comparable to the highest PCE of single‐crystalline silicon solar cells.^[^ ^2^ ^]^ Although organic electron transport layers (ETLs), such as phenyl‐C_61_‐butyric acid methyl ester (PC_61_BM), have been widely employed in PSCs, with high efficiency and minimal hysteresis, they present several limitations.^[^ ^3^, ^4^ ^]^ These limitations include non‐tunable Fermi levels, which induce charge carrier recombination with elevated series resistance, complex and costly synthesis processes, and susceptibility to environmental degradation. Moreover, expensive organic transport layers increase the cost of production, posing a significant barrier to the large‐scale commercialization of PSCs.^[^ ^5^ ^]^ Therefore, the economic and scalability challenges associated with ETLs necessitate the exploration of alternative materials that offer good performance and affordability.

Inorganic ETLs, consisting of titanium dioxide (TiO_2_), tin dioxide (SnO_2_), and zinc oxide (ZnO), have emerged as promising alternatives to address these challenges, offering tunable energy levels, enhanced stability, and cost‐effectiveness for industrial fabrication.^[^ ^6^, ^7^, ^8^, ^9^ ^]^ However, the development of high‐quality inorganic ETLs comparable to thermally evaporated C_60_ or spin‐coated PC_61_BM remains challenging, particularly because of the sensitivity of the organic perovskite layer to high‐temperature processing and energetic deposition methods. In regular PSC architectures, sol‐gel methods are widely employed to prepare high‐quality SnO_2_ or TiO_2_ layers using water baths or spin‐coating processes.^[^ ^10^, ^11^ ^]^ However, these conventional approaches face significant limitations when applied to the top surface of the perovskite layers in inverted devices. This primary challenge originates from the extreme sensitivity of perovskites to moisture, as exposure to substantial amounts of water during processing leads to the rapid decomposition of the active layer. Furthermore, high‐energy deposition techniques, such as magnetron sputtering, which are commonly used for metal oxide deposition, are unsuitable for applications on perovskite surfaces. Owing to the soft and sensitive nature of perovskite materials, they are particularly vulnerable to physical damage from energetic particle bombardment, which causes defects and limits the performance of solar cells.^[^ ^12^ ^]^ Gao et al. demonstrated significant progress in developing PSCs using atomic layer deposition (ALD)‐processed SnO_2_ as the ETLs, achieving PCEs comparable to those of conventional organic ETL‐based devices.^[^ ^13^ ^]^ The inherent environmental stability of metal oxides results in PSCs with notably superior stability compared with C_60_‐based devices, attracting considerable attention. However, this approach still relies on organic transport materials as passivation layers to provide a passivating and buffering interlayer that is compatible with ALD deposition. Moreover, the fabrication process inevitably requires the use of an organic ALD precursor, such as tetrakisdimethylamino tin (IV) (TDMASn), which incurs relatively high material costs.

ZnO, a wide‐bandgap (3.3 eV) n‐type semiconductor, offers advantages such as a high exciton binding energy, low‐cost fabrication, and high electron mobility.^[^ ^14^, ^15^ ^]^ These properties render it an attractive ETL for use in high‐performance inverted PSCs. Various strategies have been explored to optimize ZnO‐based ETLs in PSCs. Previous studies have demonstrated that doping ZnO with elements such as aluminum and gallium can significantly enhance its electrical properties and improve the overall device performance.^[^ ^16^, ^17^, ^18^ ^]^ These dopants modify the electronic structure of ZnO, resulting in increased conductivity and improved charge transport characteristics. Additionally, to address the ZnO‐induced deprotonation reactions of perovskite layers, organic isolation layers, such as polyethyleneimine ethoxylated (PEIE) layers, have been proposed as buffer barriers.^[^ ^19^, ^20^ ^]^ While these approaches have shown promise in device performance and stability, they present a significant drawback: the high cost and hydrophilic nature of the organic materials used in these isolation layers sacrifice the economic advantages inherent to inorganic transport layers.

In this study, we present a strategy that simultaneously utilizes tungsten‐doped zinc oxide (WZO) and atomically coherent interfaced SnO_2_ nanoparticles as electron extraction bilayers to mitigate the interfacial deprotonation reactions associated with ZnO. First, we employed SnO_2_ nanoparticles as an isolation interlayer to protect the perovskite surface and suppress the deprotonation reactions.^[^ ^21^, ^22^ ^]^ We then utilized thermal co‐evaporation to deposit zinc oxide and tungsten simultaneously. This approach provides substantial cost advantages over ALD‐grown SnO_2_ by obviating the requirement for expensive organic precursors, such as TDMASn.^[^ ^13^, ^23^ ^]^ Sequentially, we can finely tune the energy level of WZO by varying the tungsten content, tailoring optimal energy level alignment among the adjacent interface layers. This multiple‐ETL configuration exhibits superior electrical properties, including low resistivity and increased carrier mobility, collectively improving charge transport in PSCs. Our innovative approach yielded the champion PCE in inverted PSC with an all‐inorganic ETL, demonstrating high performance with negligible hysteresis and high stability of continuous operation at the maximum power point.

## 2 Results and Discussion

**Figure 1:**
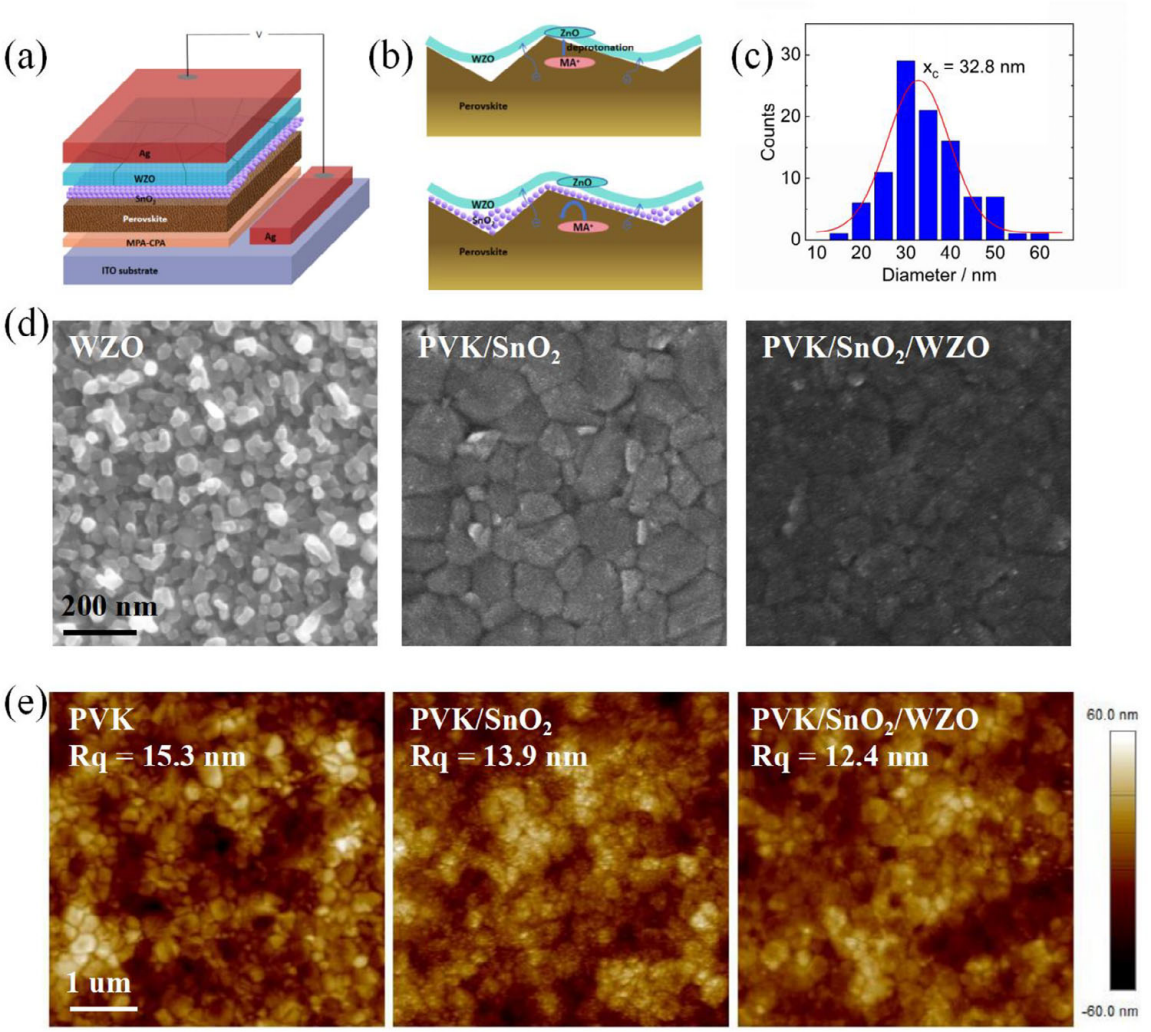
a) Schematic illustration of PSCs. b) Schematic illustration of passivation and inhibiting deprotonation reactions in ETLs. c) Grain size distribution diagram for WZO by thermal evaporation. d) SEM spectra of WZO, perovskite/SnO_2_, and perovskite/SnO_2_/WZO surface. e) AFM spectra of perovskite, perovskite/SnO_2_, and perovskite/SnO_2_/WZO surface.

In an inverted PSC structure, the hole transport layer (HTL) is composed of 2‐(4‐(Bis(4‐methoxyphenyl)amino)phenyl)‐1‐cyanovinyl)phosphonic acid (MPA‐CPA). The light‐absorbing layer consisted of two types of perovskite with the nominal composition Cs_0.05_FA_0.9_MA_0.05_Pb(I_0.95_Br_0.05_)_3_ and Cs_0.05_FA_0.95_PbI_2.94_Br_0.06_, where FA represents formamidinium and MA represents methylammonium, featuring band gaps of 1.58 and 1.54 eV, respectively. The ETL was a novel composite consisting of tin oxide nanoparticles and tungsten‐doped zinc oxide. Indium tin oxide (ITO) and silver were used as electrodes (**Figure** **1**a).

The use of multiple ETLs in PSCs offered several distinct advantages that enhance the device performance without affecting perovskite crystallization (Figure 1b; Figure S1, Supporting Information). Thermal evaporation of WZO with an adjustable bandgap allowed for precise energy‐level alignment with the perovskite layer, facilitating efficient electron extraction. Additionally, spin‐coated SnO_2_ nanoparticles played a crucial role in passivating inherent defects on the perovskite surface, thereby improving the surface morphology and reducing recombination losses. The mismatch in lattice sizes between WZO and perovskite can lead to lattice distortion when in direct contact, whereas the SnO_2_ layer effectively mitigates this issue by better matching the lattice constants. Furthermore, the SnO_2_ layer acts as a physical barrier, isolating ZnO from the perovskite and inhibiting the migration of methylamine ions, which can result in deprotonation.^[^ ^24^ ^]^ This strategic combination of materials not only optimized charge transport but also enhanced the overall stability and efficiency of inverted PSCs.

Scanning electron microscopy (SEM) images provided valuable insights into the morphology of the thermally evaporated WZO layer. SEM confirmed that the WZO film adopted a granular morphology, displaying an average particle size of 32.8 nm, uniformly and densely covering the ITO substrate (Figure 1c,d). This uniform coverage indicates high‐quality deposition. On the perovskite substrate, the spin‐coated SnO_2_ layer was nearly imperceptible because of its extremely fine particle size, which contributed to minimal surface disruption. Notably, the WZO layer demonstrated a higher conductivity than both the perovskite and SnO_2_ layers (Table S1, Supporting Information). Consequently, the areas covered by WZO appeared darker in the SEM images, resulting in an overall dark and uniform appearance. This uniformity underscores the effectiveness of the WZO layer in providing a consistent and conductive interface, which is crucial for enhancing the performance of the inverted PSCs. Atomic force microscopy (AFM) analysis provided further evidence for the passivation effect of the SnO_2_ layer and the densification achieved by the WZO layer in the PSC structure (Figure 1e). The root‐mean‐square (*Rq*) roughness of the pristine perovskite surface was 15.3 nm. Upon the addition of the SnO_2_ layer, the *R_q_* value decreases to 13.9 nm, indicating an effective smoothing of the perovskite layer. A further reduction in *R_q_* value to 12.4 nm was observed with the incorporation of the WZO layer, suggesting enhanced densification and uniformity of the surface.

**Figure 2:**
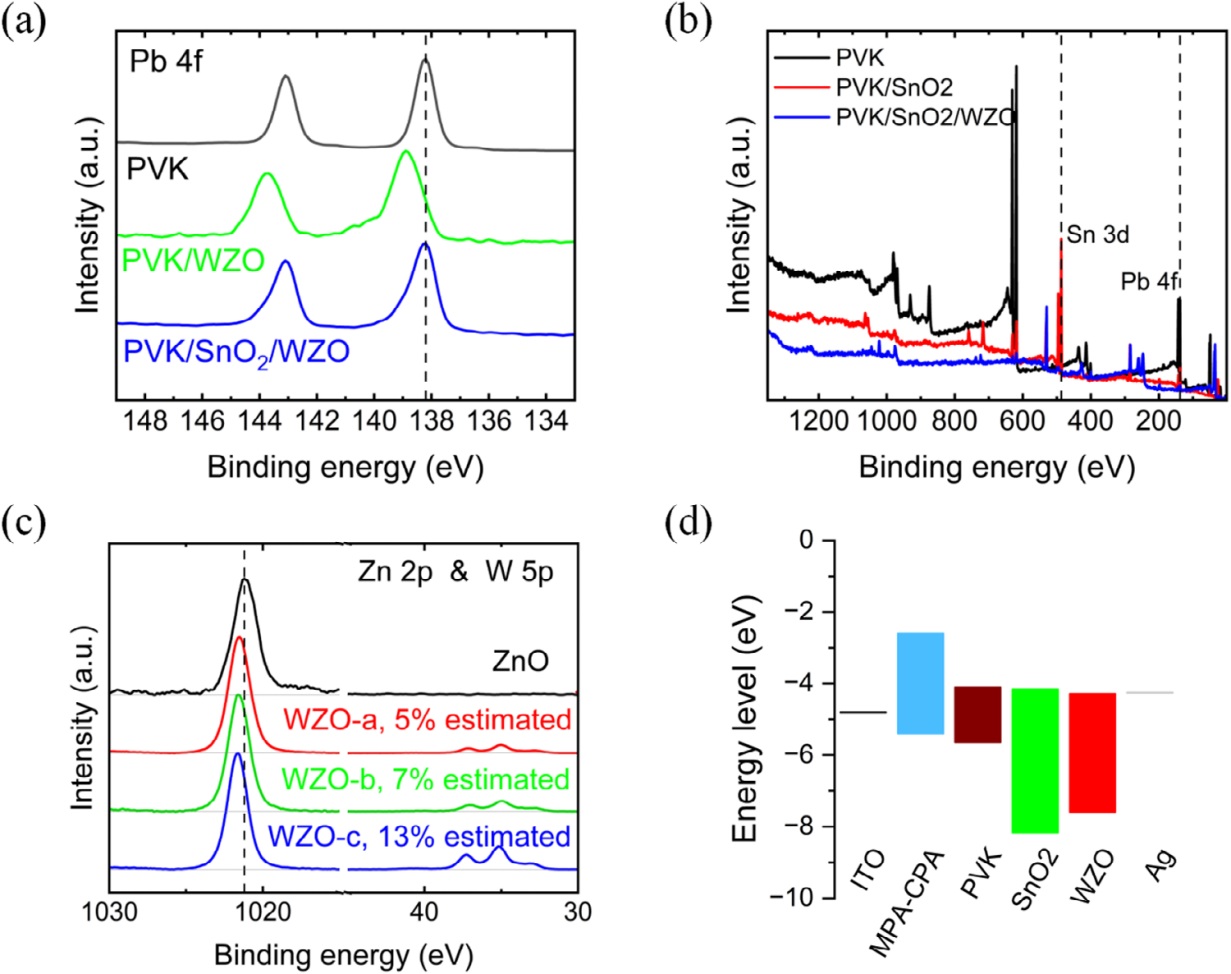
Bounding energy of a) Pb 4f of perovskite, perovskite/WZO, and perovskite/SnO_2_/WZO pieces, normalized by 138.2 eV peak height, b) overview of the elemental distribution of perovskite, perovskite/SnO_2_, perovskite/SnO_2_/WZO pieces, without any normalization, and c) Zn 2p and W 5p of ZnO nanoparticles, WZO‐a, WZO‐b, and WZO‐c (16, 18, 20 Watts of thermal evaporation, respectively), normalized by Zn 2p peak height, by high‐resolution XPS core‐level spectra. d) Energy alignment of functional layers extracted from UPS measurements.

X‐ray photoelectron spectroscopy (XPS) analysis provides critical insights into the interfacial interactions. Upon deposition of the WZO layer on the perovskite surface, an increase in the binding energy of Pb 4f was observed (**Figure** **2**a). This shift suggests that a deprotonation reaction occurred between ZnO and the perovskite material. In contrast, when a layer of SnO_2_ was introduced between the perovskite surface and the WZO, the Pb 4f binding energy remained similar to that of the pristine perovskite surface. This finding demonstrates that the SnO_2_ layer successfully suppressed deprotonation reactions. The ability of SnO_2_ to prevent such interfacial reactions underscores its role as a protective barrier that maintains the integrity of the perovskite layer. Figure 2b provides an overview of the elemental distribution of the three distinct profiles: perovskite (PVK in the figures), perovskite/SnO_2_, and perovskite/SnO_2_/WZO. Upon deposition of SnO_2_ on the perovskite surface, a significant suppression of the Pb peaks was observed, indicating effective coverage by the SnO_2_ layer. This suggests that SnO_2_ formed a uniform and dense layer over the perovskite, which is crucial for passivating surface defects and preventing undesirable reactions. Further deposition of the WZO layer resulted in the near‐disappearance of both the Pb and Sn peaks. This indicated the high density and uniformity of the WZO layer, which effectively encapsulated the underlying layers.

Figure 2c presents the XPS analysis of WZO with varying tungsten contents, including pure ZnO. The Zn 2p peaks shifted as the W content increased. This upward shift in the binding energy may be attributed to the formation of zinc tungstate and an increase in oxygen vacancies induced by high‐temperature processing.^[^ ^15^, ^25^, ^26^ ^]^ Previous studies have indicated that the oxygen vacancies in ZnO are a primary factor contributing to the conductivity of the material. Furthermore, by comparing the area ratios of the Zn peaks to the W peaks, we observed a progressive increase in W content with changes in the deposition method of the ZnO or WZO layers. Notably, no W peaks were detected for the spin‐coated ZnO nanoparticles. In the WZO deposited at 16 W, ≈5% of tungsten was detected in the calculated areas of the XPS peaks. This increased to 7% estimated at 18 Watts and further to 13% estimated at 20 Watts deposition. These findings suggest that the incorporation of W into the ZnO matrix greatly depends on the deposition conditions, which in turn affect the electronic properties of the material. Adjusting the tungsten content via the deposition parameters enables the precise modulation of the conductivity and electronic properties of WZO, thereby optimizing the performance of perovskite solar cells. Figure S2 (Supporting Information) illustrates the energy‐level alignments achieved through Fermi‐level alignment in the WZO layers with varying tungsten contents. The data were collected using ultraviolet photoelectron spectroscopy (UPS). The valence band maximum (VBM) of the WZO layers increased compared with that of ZnO, indicating a shift in the semiconductor's characteristics and suggesting that the material's electronic properties of the material can be finely tuned by adjusting the tungsten content. According to previous research, the incorporation of higher valence state tungsten into zinc oxide can provide additional free electrons.^[^ ^18^ ^]^ Specifically, when each W^6+^ ion substitutes for a Zn^2+^ ion in the ZnO lattice, it introduces additional electrons into the crystal structure. These excess electrons act as free carriers, significantly increasing the carrier concentration in ZnO and improving the electron transport properties in WZO. Hence, the tungsten doping can improve the performance of solar cells, especially the fill factors, by enhancing the conductivity of zinc oxide.

Figure 2d shows an energy‐level diagram of the complete PSC device architecture. The band gap for perovskites and WZO was obtained from the absorption spectra (Figures S3–S5, Supporting Information), and the key parameters are listed in Table S3 (Supporting Information), together with the work function and valence band (Figures S6 and S7, Supporting Information). The incorporation of SnO_2_ creates a stepwise energy‐level alignment between the perovskite and WZO layers, effectively minimizing voltage losses at the interfaces. Notably, SnO_2_ exhibited a deeper VBM than WZO, enabling more efficient hole‐blocking capabilities. This enhanced charge selectivity contributes significantly to an improvement in the PCE of solar cells.

**Figure 3:**
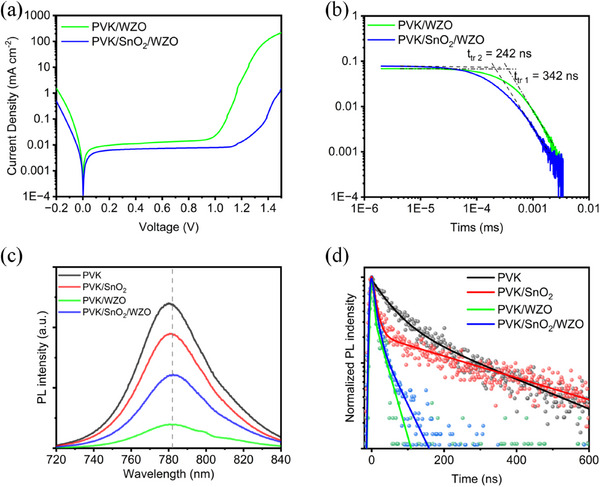
a) Dark *J*–*V* curves and b) electron mobility by FlyTOF obtained from PSCs fabricated with and without SnO_2_. c) Steady‐state PL spectra and d) time‐resolved photoluminescence properties upon 400‐nm laser pulses excitation of the perovskite, perovskite/SnO_2_, perovskite/WZO, and perovskite/SnO_2_/WZO specimens.

Dark‐current measurements indicated a markedly reduced dark current in the SnO_2_‐incorporated devices relative to the perovskite/WZO devices, indicating superior device quality and reduced recombination losses (**Figure** **3**a). To further investigate the charge‐transport dynamics, we employed time‐of‐flight (TOF) measurements to quantify the electron mobility (Figure 3b). Owing to the thickness requirements of the TOF measurements, we used semi‐complete devices, specifically ITO/perovskite/SnO_2_/WZO/Ag and ITO/perovskite/WZO/Ag, to compare the differences in the carrier mobility before and after the addition of the SnO_2_ layer. The results demonstrate that the PSCs with the SnO_2_ layer showed a notably reduced carrier transit time t*_tr_*, decreasing from 342 to 242 ns, compared with that of devices without SnO_2_. Consequently, the calculated electron mobility increased from 4.67×10^−2^ to 6.78×10^−2^ cm^2^ V^−1^ s^−1^. This enhancement in the carrier mobility provides strong evidence that the SnO_2_ brings out an atomically coherent interlayer above the perovskite bulk and effectively passivates the perovskite surface, significantly reducing the interface defects that would otherwise arise from the reaction between the perovskite and ZnO.^[^ ^27^ ^]^

Photoluminescence (PL) studies provided additional clarity regarding the interfacial interactions within the perovskite solar cells (Figure 3c). The PL intensity was measured from the surface of the perovskite layer. When the perovskite surface was covered with the ETL, a reduction in the fluorescence intensity was observed. Notably, the perovskite film covered solely with WZO exhibited the lowest fluorescence intensity. This reduction can be attributed to the deprotonation of ZnO, which disrupted the perovskite morphology and affected its optical properties. Comprehensive SEM, AFM, and XPS analyses already demonstrated the dense coverage provided by the SnO_2_ layer. Additionally, Figure S8 (Supporting Information), showing the energy dispersive X‐ray spectroscopy (EDS) analysis results, indicated that SnO_2_ did not aggregate on the perovskite surface, thereby maintaining uniform coverage. Therefore, the complete perovskite/SnO_2_/WZO device structure can be considered to exhibit better charge‐carrier extraction capability. The reduced PL intensity in the presence of WZO suggests efficient quenching of the photogenerated excitons, which is indicative of enhanced charge separation and extraction. This efficient quenching is crucial for improving the overall efficiency of solar cells, as it minimizes recombination losses and maximizes the photocurrent.

Time‐resolved photoluminescence (TRPL) was employed as a complementary technique to the PL experiments to obtain more data on charge carrier transport (Figure 3d; Table S3, Supporting Information). The measurements were performed on half‐devices containing hole transport layers. The effective lifetimes for pure perovskite, devices with SnO_2_, devices with WZO, and devices with SnO_2_/WZO were 76.10, 22.36, 10.24, and 15.48 ns, respectively. Based on the comparison of luminescence intensity and effective lifetime among several samples, it is evident that SnO_2_, WZO, and SnO_2_/WZO can all extract photogenerated carriers from the perovskite. Among these, WZO shows the strongest luminescence quenching and lifetime reduction, primarily due to its larger conduction band energy difference to the perovskite and interface defect recombination caused by interfacial reactions.^[^ ^28^ ^]^ The electron transfer rate at the film interface is largely determined by the conduction band offset, which serves as the driving force. Generally, a larger conduction band offset enhances the driving force for electron transfer, thereby increasing the transfer rate, up to a certain limit.^[^ ^29^ ^]^ While SnO_2_ can also effectively extract photogenerated electrons from the perovskite, its conduction band is closer to that of the perovskite, resulting in a smaller electron transfer driving force. Additionally, according to previous literature reports, SnO_2_‐perovskite interface self‐passivation was found,^[^ ^30^ ^]^ thereby reducing interface recombination. Therefore, compared to WZO, SnO_2_ exhibits less quenching of perovskite luminescence intensity and less reduction in luminescence lifetime.

**Figure 4:**
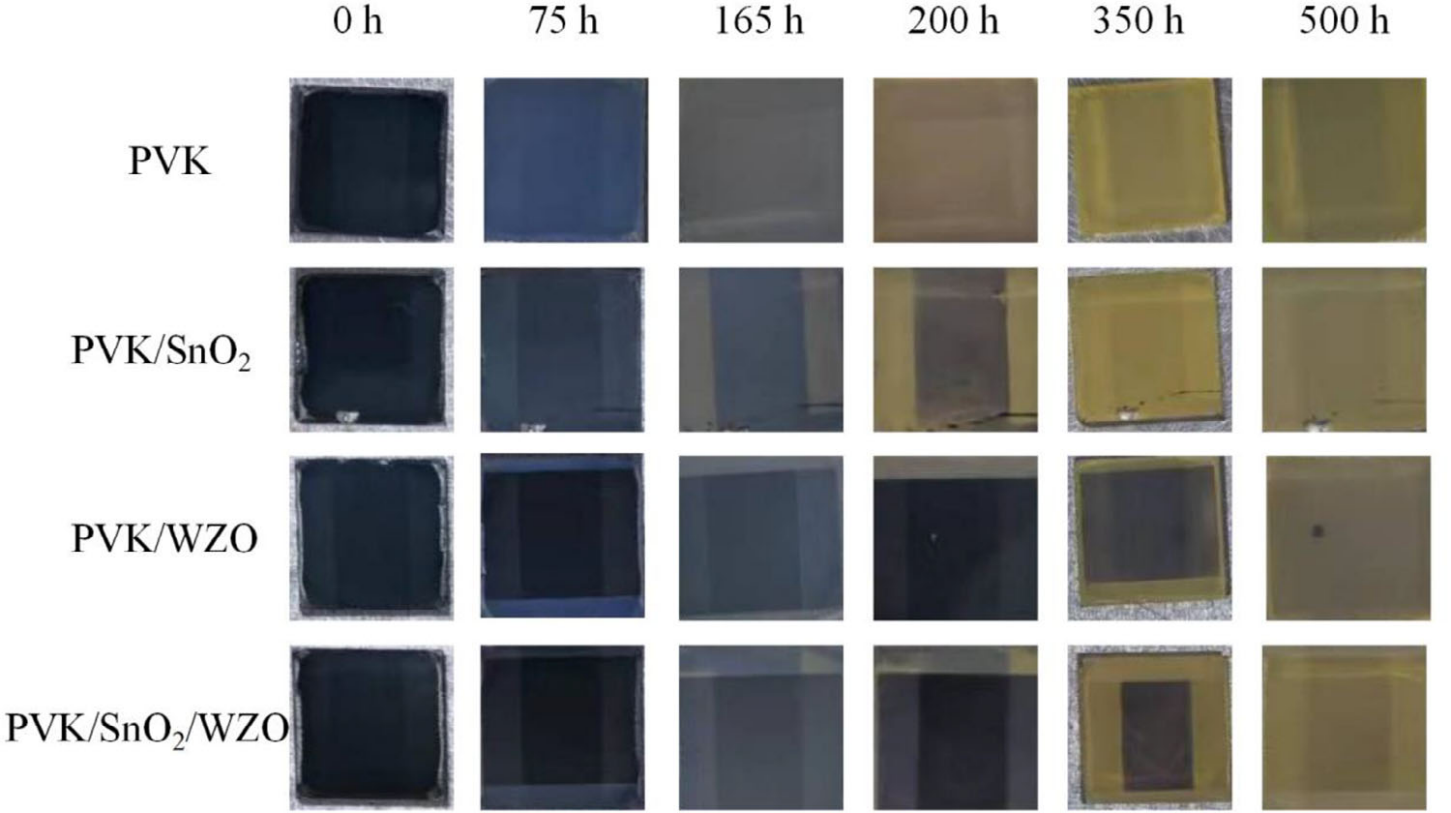
Stability of unencapsulated perovskite, perovskite/SnO_2_, perovskite/WZO, and perovskite/SnO_2_/WZO specimens exposed to humid air at 85 °C, with humidity levels ranging from 70% to 100%.

A decomposition experiment was conducted to visually assess the stability of perovskite solar cells under harsh environmental conditions (**Figure** **4**). Unencapsulated devices were exposed to humid air at 85 °C, with humidity levels ranging from 70% to 100%, to determine the time required for degradation. Devices without a transport layer on the surface exhibited rapid discoloration within 165 h, indicating the onset of decomposition. In contrast, devices coated with spin‐coated SnO_2_ exhibited improved stability, delaying the degradation process. Remarkably, the devices with thermally evaporated WZO maintained their alpha‐phase stability for up to 350 h. The observed decomposition arose from the thermal evaporation process, whereas the uneven substrate offered inadequate protection. The superior performance of the WZO‐coated devices suggests that the dense and uniform coverage provided by the WZO effectively protects the perovskite layer from moisture and thermal stress.

**Figure 5:**
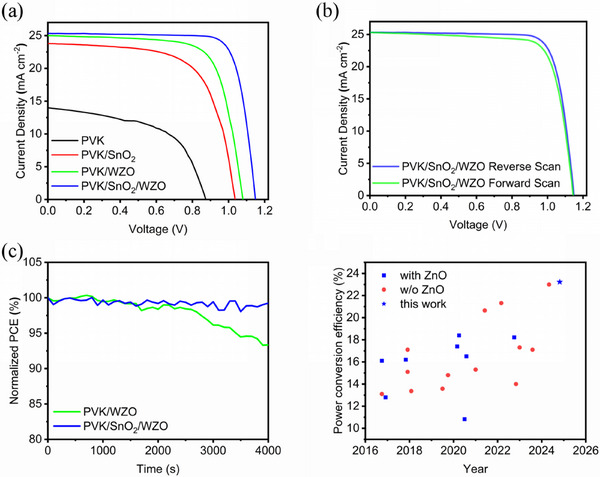
Performances of a) perovskite, perovskite/SnO_2_, perovskite/WZO, and perovskite/SnO_2_/WZO devices, b) forward scan and reverse scan, c) continuous operation at their maximum power point under simulated AM1.5 illumination, and d) summary of all‐inorganic ETL in inverted PSCs.^[^ ^23^, ^31^, ^32^, ^33^, ^34^, ^35^, ^36^, ^37^, ^38^, ^39^, ^40^, ^41^, ^42^, ^43^, ^44^, ^45^, ^46^, ^47^, ^48^, ^49^ ^]^

**Figures** **5** and S9 (Supporting Information) illustrate the photovoltaic performances of the PSCs. Devices incorporating only the SnO_2_ layer demonstrated better performance than those without any ETLs, consistent with our carrier mobility analysis, confirming the inherent capability of SnO_2_ as an electron transport material. More significantly, compared with devices with WZO alone, the incorporation of SnO_2_ as a passivation and isolation layer to prevent deprotonation reactions led to remarkable performance improvements. The champion device achieved a PCE of 23.19%, featuring an open‐circuit voltage of 1.15 V, short‐circuit current density of 25.3 mA cm^−2^, and fill factor of 79.7%, with negligible hysteresis (Figure 5b; Tables S4 and S5, Supporting Information), which is a new record for PSCs with all‐inorganic ETLs. The fabrication method exhibited excellent reproducibility, supported by consistent photovoltaic parameters across 15 devices (Figure S10, Supporting Information). Furthermore, the SnO_2_‐WZO architecture demonstrated applicability across two perovskite compositions (Figure S11, Supporting Information). The external quantum efficiency spectra (Figure S12, Supporting Information) demonstrated that the integrated short‐circuit current densities closely matched the values obtained from the *J*–*V* measurements. Maximum power point tracking revealed superior operational stability in devices with a SnO_2_ isolation layer compared with those without. After 4000 s of continuous operation, the devices incorporating SnO_2_ maintained 99.2% of their initial PCE, whereas the devices without SnO_2_ retained only 93.3% of their initial PCE (Figure 5c). This significant difference in stability can be attributed to continuous deprotonation reactions and existing defects, leading to perovskite decomposition in devices lacking a SnO_2_ layer. This improved stability underscores SnO_2_’s pivotal role in mitigating interfacial degradation and sustaining the device performance during prolonged operation.

## 3 Conclusion

In this study, we demonstrated a novel approach for fabricating high‐performance inverted PSCs utilizing all‐inorganic ETLs. The strategic combination of SnO_2_ and WZO addresses multiple challenges in the PSC design. The SnO_2_ layer effectively passivated the surface defects and prevented detrimental deprotonation reactions between the perovskite and ZnO, whereas the WZO layer, with its tunable energy levels through controlled tungsten doping, facilitated efficient charge extraction. This dual‐layer architecture achieved a remarkable power conversion efficiency of 23.19% with negligible hysteresis, representing the highest reported efficiency for inverted PSCs using all‐inorganic ETLs. Furthermore, the devices exhibited exceptional operational stability, maintaining 99.2% of their initial PCE after 4000 s of continuous operation at the maximum power point. This cost‐efficient, fully inorganic strategy bypasses the use of expensive organic materials and represents a viable route for industrial‐scale perovskite solar cell production.

## 4 Experimental Section

The solar cell devices were composed of ITO/MPA‐CPA/perovskite/SnO_2_/WZO/Ag. For the perovskite Cs_0.05_FA_0.9_MA_0.05_PbI_2.85_Br_0.15_, a higher proportion (9 mol%) of PbI_2_ was added in 1.5 mol mL^−1^ precursor. For the perovskite Cs_0.05_FA_0.95_PbI_2.94_Br_0.06_, a higher proportion (10 mol%) of PbI_2_ was added in 1.55 mol mL^−1^ precursor. In addition, MACl (Methylamine hydrochloride, 10 mol%) and PPABr (phenylpropylamine bromide, 0.16 mol%) were used as additive passivation constituents in the perovskite precursors for both kinds. In detial, for the 1.5 mol mL^−1^ Cs_0.05_FA_0.9_MA_0.05_PbI_2.85_Br_0.15_ perovskite, 718.32 mg of lead iodide (an additional 9% as mentioned, for high‐efficiency devices), 28.21 mg of lead bromide, 19.49 mg of cesium iodide, 232.46 mg of formamidinium iodide, 7.97 mg of methylammonium bromide, 15.19 mg of methylamine hydrochloride (10 mol% as stated), and 0.8 mg of phenylpropylamine bromide (0.16 mol% as stated) were used. For the 1.55 mol mL^−1^ Cs_0.05_FA_0.95_PbI_2.94_Br_0.06_ perovskite, 762.44 mg of lead iodide (an additional 10% as mentioned, for high‐efficiency devices), 17.07 mg of lead bromide, 20.13 mg of cesium iodide, 253.23 mg of formamidinium iodide, 10.99 mg of methylamine hydrochloride (10 mol% as stated), and 0.8 mg of phenylpropylamine bromide (0.16 mol% as stated) were used. All solution weighing, dissolution, and spin‐coating procedures were carried out in a nitrogen‐filled glovebox. After preparation, the solutions were thoroughly mixed using a vortex mixer for at least 3 h and used within 24 h. The ITO substrates were cleaned by sequential sonication in detergent solution, deionized water, ethyl alcohol, acetone, and isopropyl alcohol (IPA) for 15 min each and placed in a drying oven for at least 3 h. Prior to preparation, the substrates were treated with UV light for 15 min. For the HTL, MPA‐CPA (35‐µL solution of 1 mg mL^−1^) in DMF was spin‐coated at 4000 rpm for 30 s and annealed at 100 °C for 10 min. The perovskite precursor solution was prepared using a 5:1 volumetric ratio of DMF to DMSO. The perovskite solution (60 µL) was spin‐coated at 4000 rpm for 40 s. At the 34th second in the whole setting, a mixed solution containing 90% CB and 10% IPA (100 µL, 0.1 mg mL^−1^ PPABr included) was uniformly deposited onto the substrate. Afterward, the perovskite substrate was annealed at 100 °C for 35 min. The SnO_2_ nanoparticles in IPA solution were dynamically spun on the perovskite at 4000 rpm for 30 s and annealed at 100 °C for 30 min. The WZO layer was thermally deposited with a controlled power under a specific vacuum condition (<1 × 10^−5^ Torr). Both the size of zinc oxide particles and the vacuum level during the evaporation process could influence the minimum power required for the onset of deposition. Specifically, smaller particle sizes and higher vacuum levels could reduce the required power. Correspondingly, experimental experience indicated that WZO exhibits similar properties under the same evaporation power. Therefore, in the article, the correlation between the final evaporation power and the test data was only presented. The optimized solar cell device was obtained under conditions of ≈1.5 mm particle size and a vacuum level of ≈2 × 10^−6^ Torr. The ZnO layer in the XPS part was spin‐coated on ITO glass at 4000 rpm for 30 s and annealed at 100 °C for 30 min. Finally, a 100‐nm‐thick Ag electrode was thermally deposited.

## Conflict of Interest

The authors declare no conflict of interest.

## Supporting information



Supporting Information

Supporting Information

## Data Availability

The data that support the findings of this study are available in the supplementary material of this article.
