# Supplementary material for: Efficient Inverted Perovskite Solar Cells Utilizing Inorganic Composite Multiple Electron Transport Layers
Source: Small. 2025 Jul 25;21(36):e11978. doi: 10.1002/smll.202411978 (PMC12423922; doi:10.1002/smll.202411978)
Supplement: Supplementary file 1 — Supporting Information [file SMLL-21-e11978-s001.docx]

Supporting Information

Efficient Inverted Perovskite Solar Cells Utilizing Inorganic Composite Multiple Electron Transport Layers

A. Zhu, H. Gu, W. Li, J. Guo, S. Li, G. Wang, J. Xia, C. Liang, S. Chen, G. Xing*


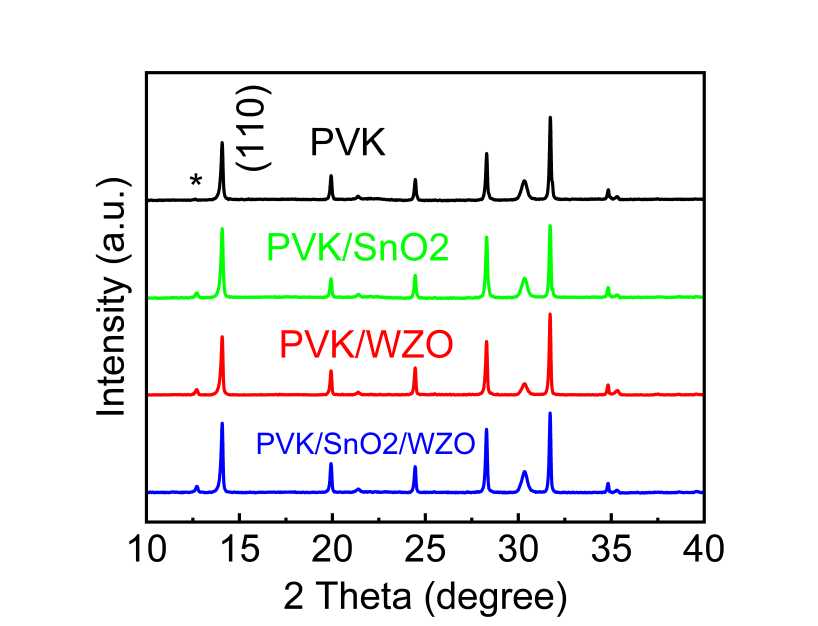


**Figure S1.** XRD images of PVK, PVK/SnO_2_, PVK/WZO, PVK/SnO_2_/WZO pieces.


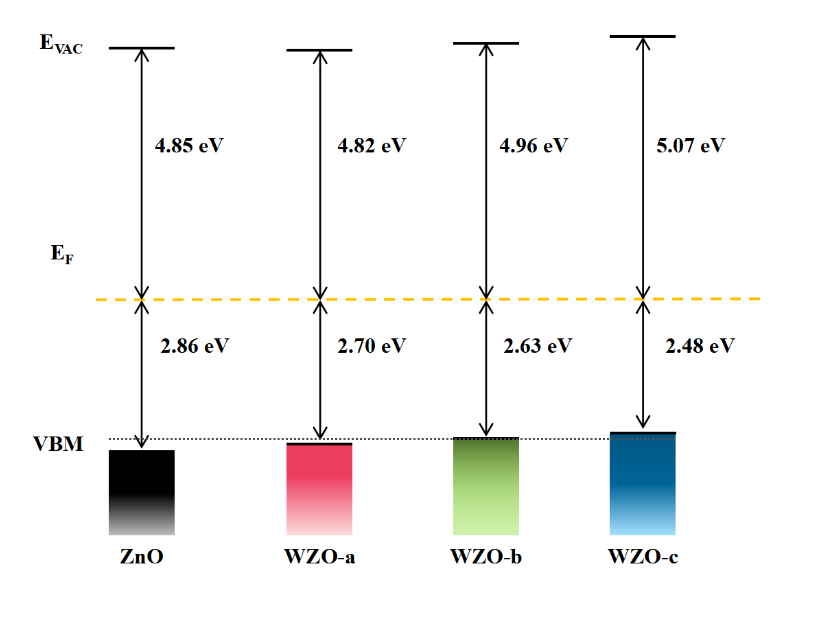


**Figure S2.** Band edge positions of functional layers extracted from UPS measurements of ZnO nanoparticles, WZO-a, WZO-b, and WZO-c. *E_VAC_* is the vacuum level, *E_F_* is the Fermi level, and VBM is the valence band maximum.


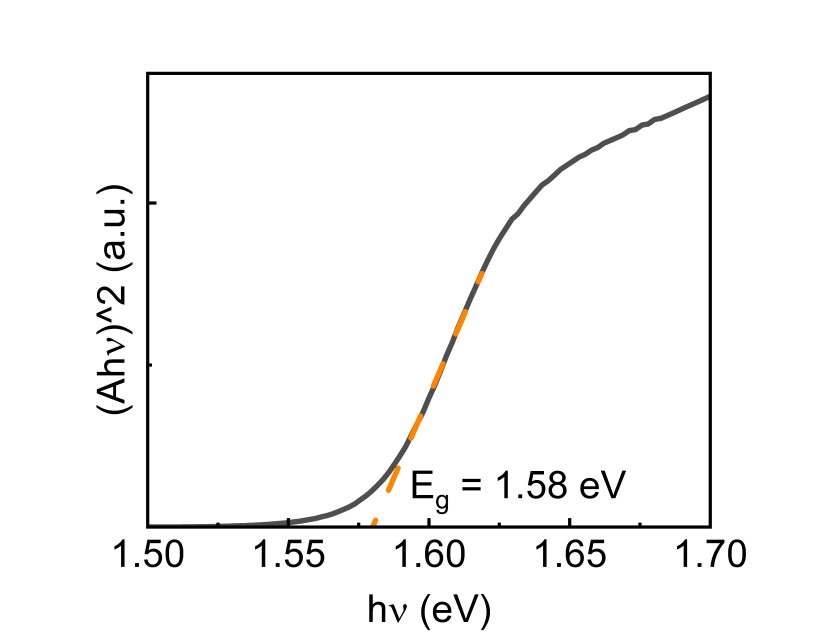


**Figure S3.** Energy gap got from absorption for perovskite Cs_0.05_FA_0.9_MA_0.05_PbI_2.85_Br_0.15_.


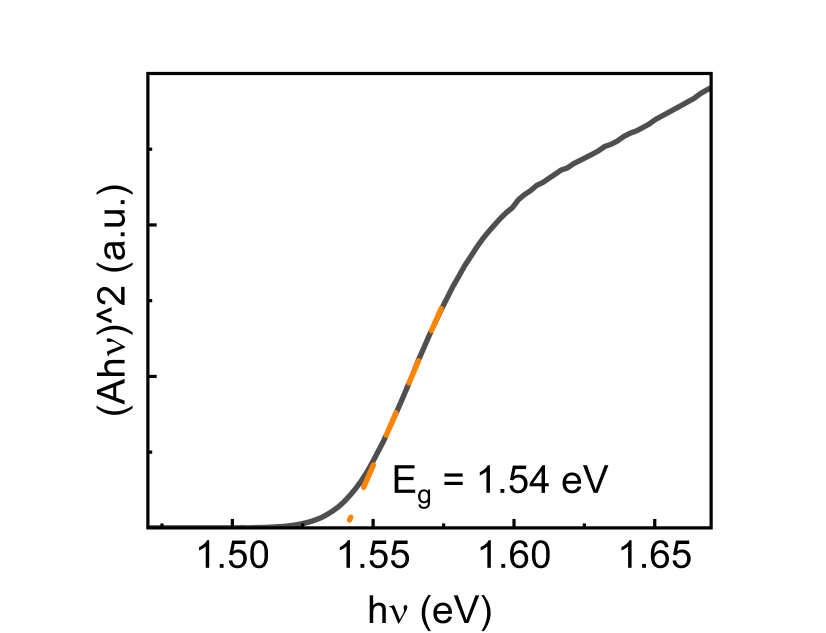


**Figure S4.** Energy gap got from absorption for perovskite Cs_0.05_FA_0.95_PbI_2.94_Br_0.06_.


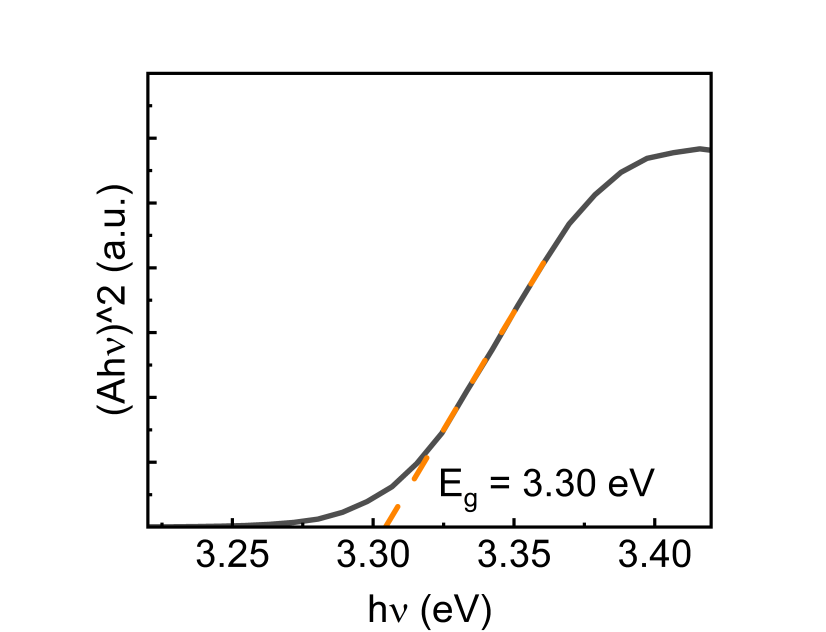


**Figure S5.** Energy gap got from absorption for WZO-b.


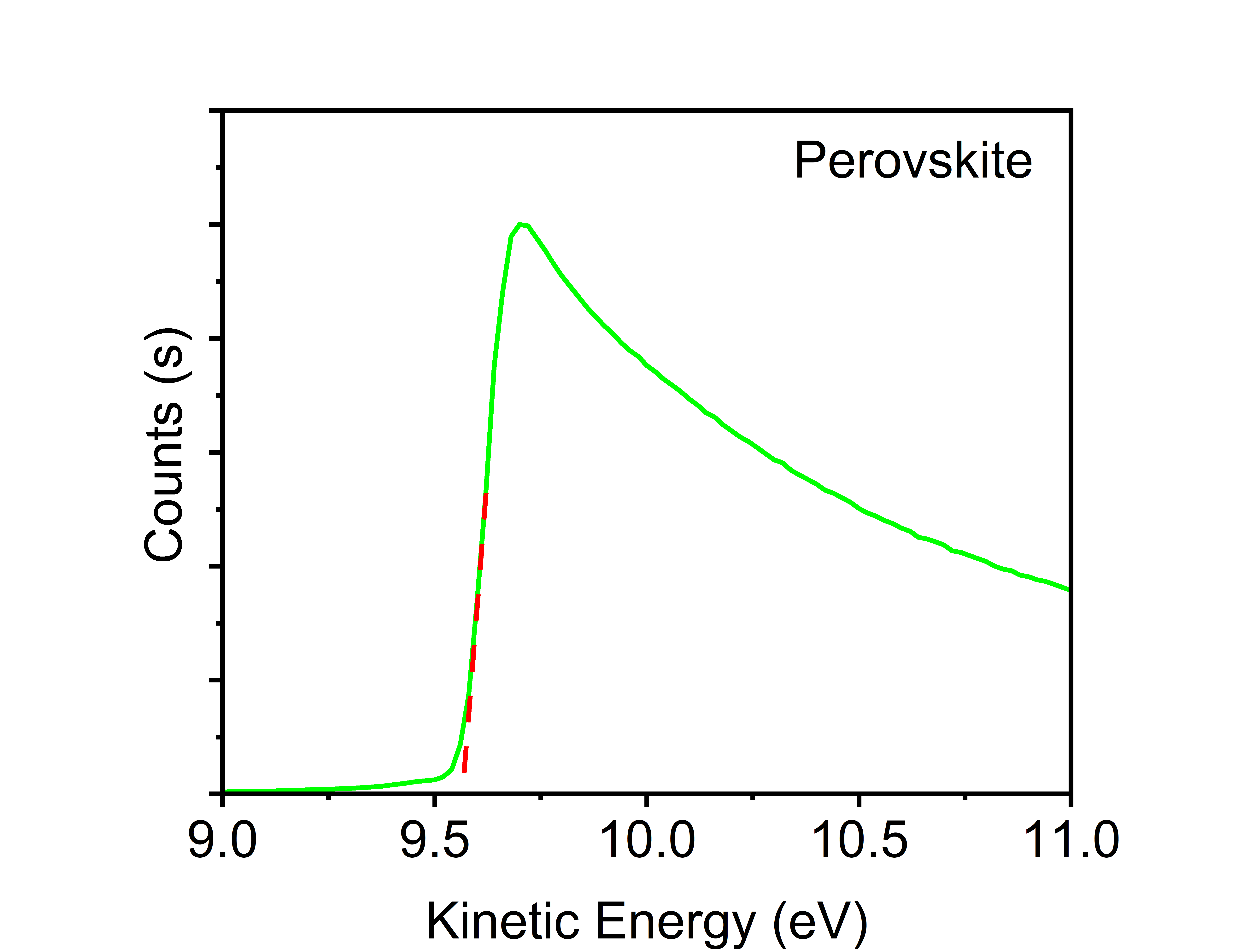


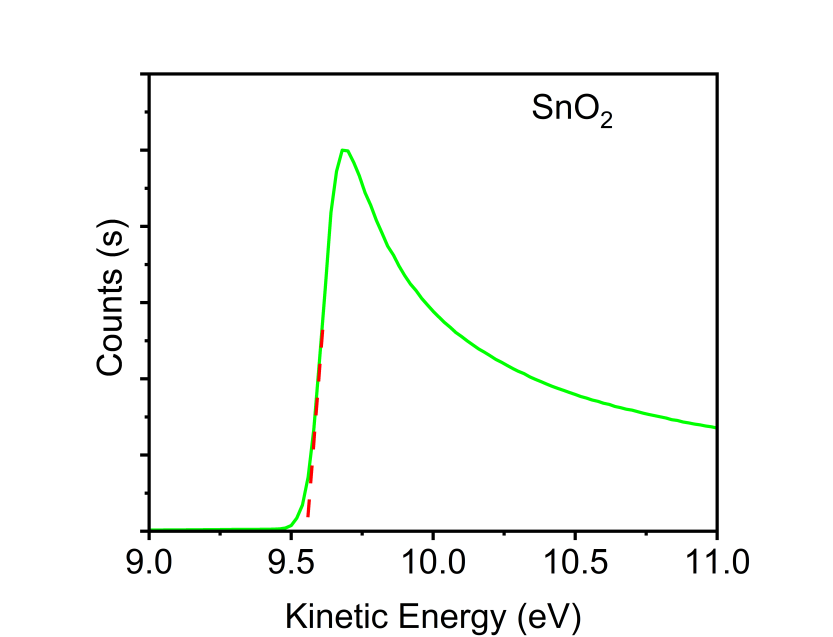


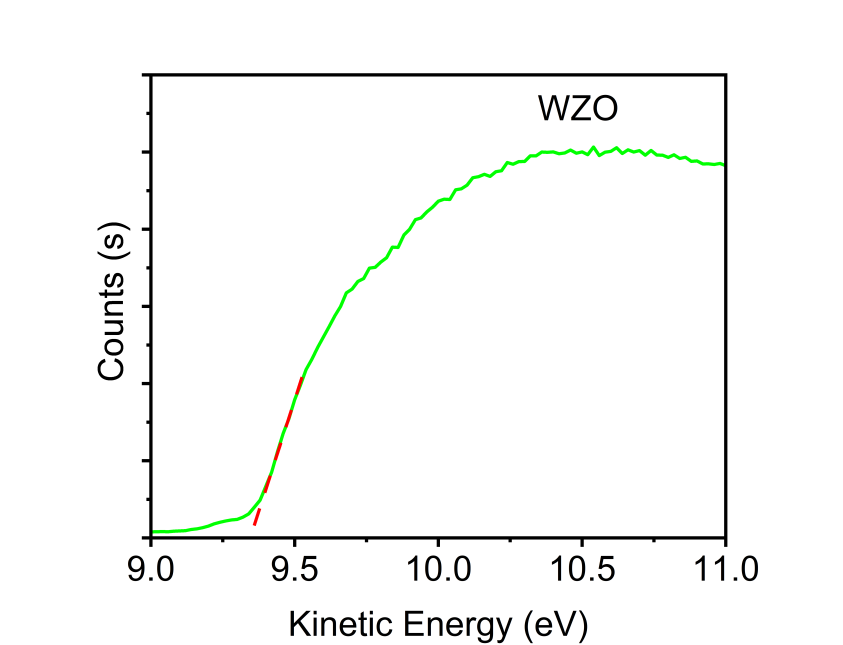


**Figure S6.** Work function by ultra-violet photoelectron spectra of different surfaces with the bias voltage of -5 V. (a) glass/ITO/MPA-CPA/Perovskite. (b) glass/ITO/MPA-CPA/Perovskite/SnO_2_. (c) glass/ITO/MPA-CPA/Perovskite/SnO_2_/WZO.


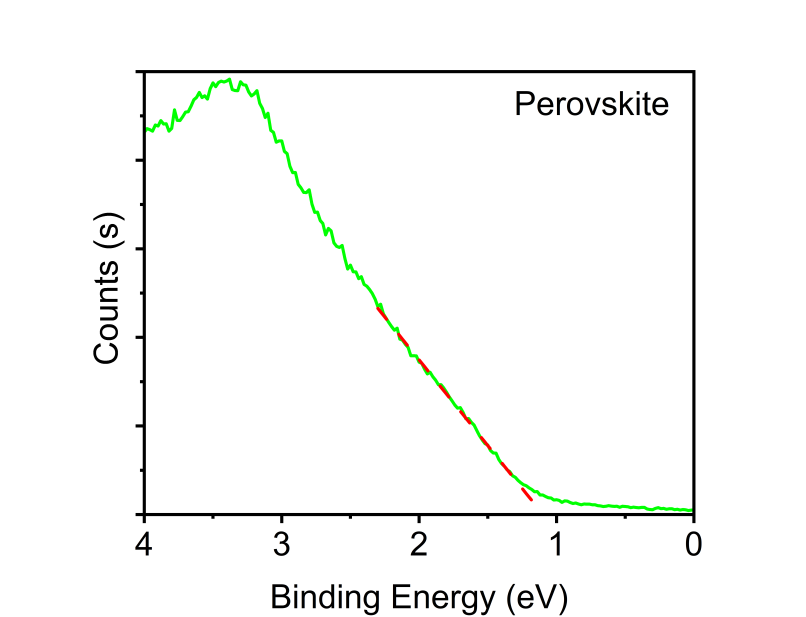


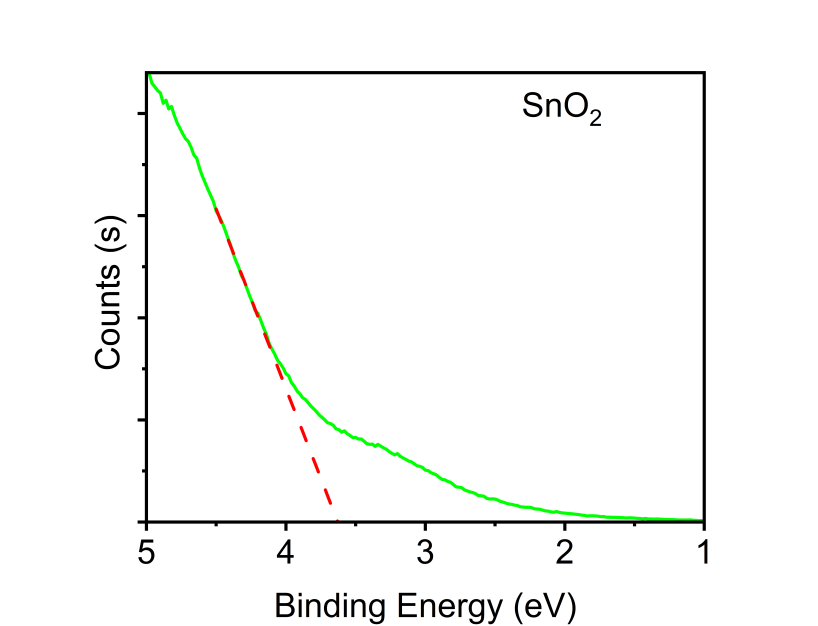


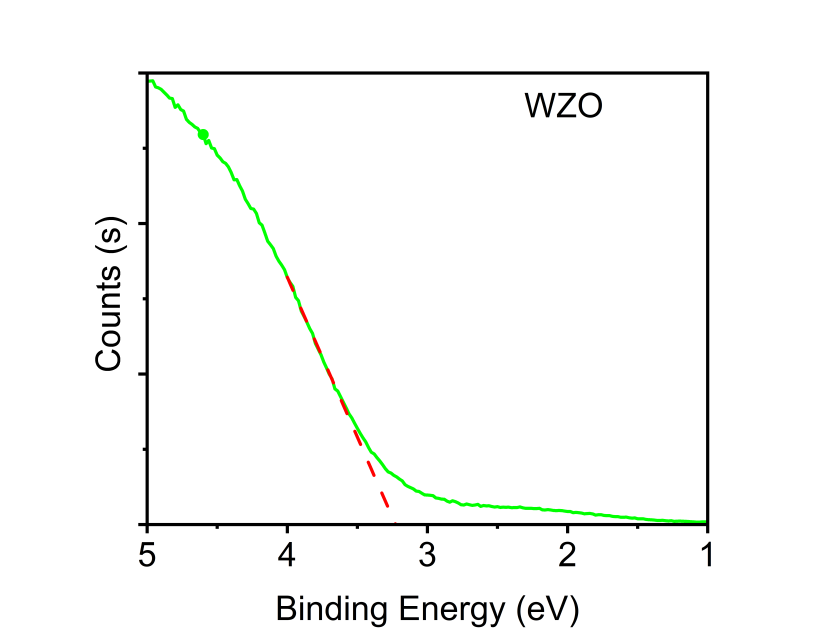


**Figure S7.** Valence band onset values by ultra-violet photoelectron spectra of different surfaces. (a) glass/ITO/MPA-CPA/Perovskite. (b) glass/ITO/MPA-CPA/Perovskite/SnO_2_. (c) glass/ITO/MPA-CPA/Perovskite/SnO_2_/WZO.


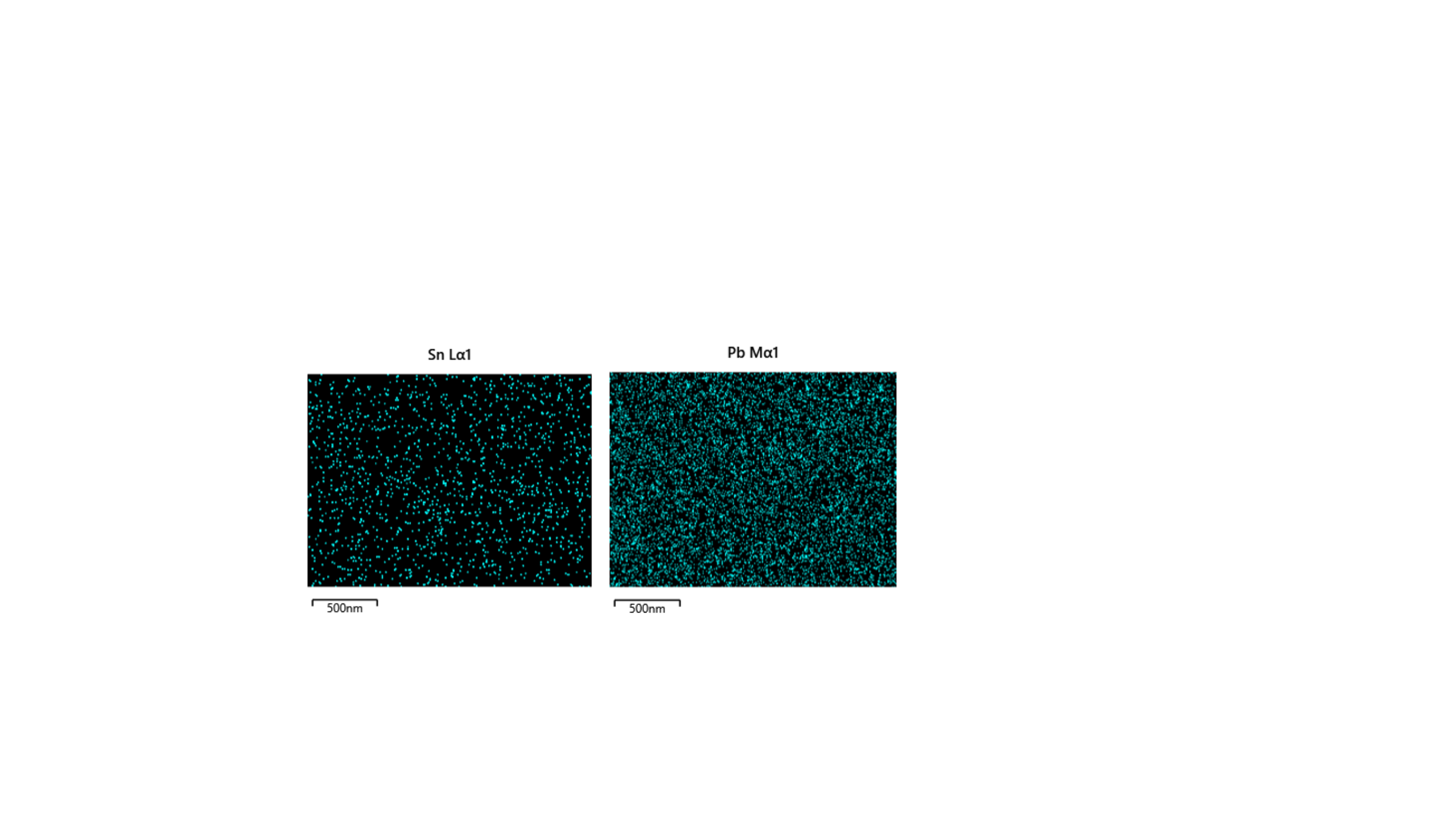


**Figure S8.** EDS spectra for perovskite/SnO_2_ piece of Sn elements and Pb elements.


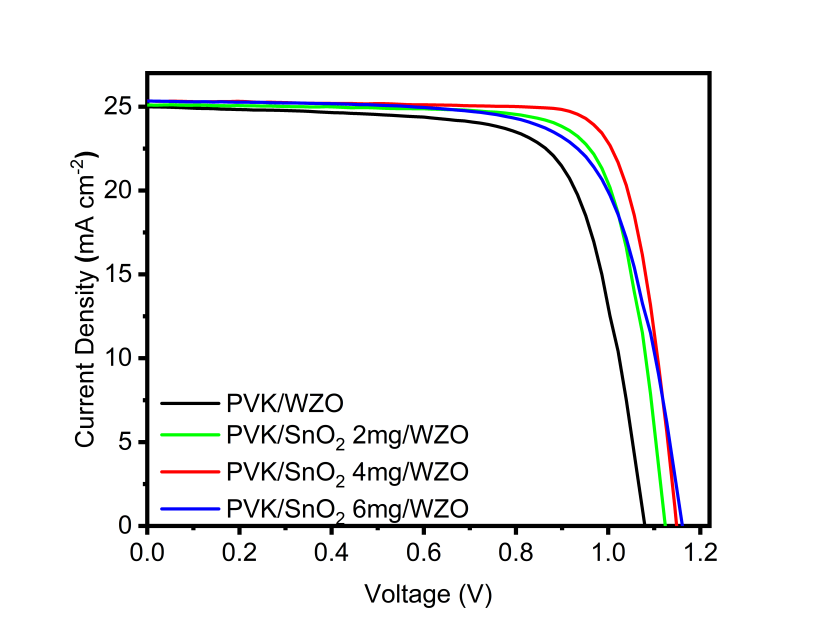


**Figure S9.** Performance for different concentration of spin-coated tin oxide nanoparticles by PVK/SnO_2_/WZO devices.


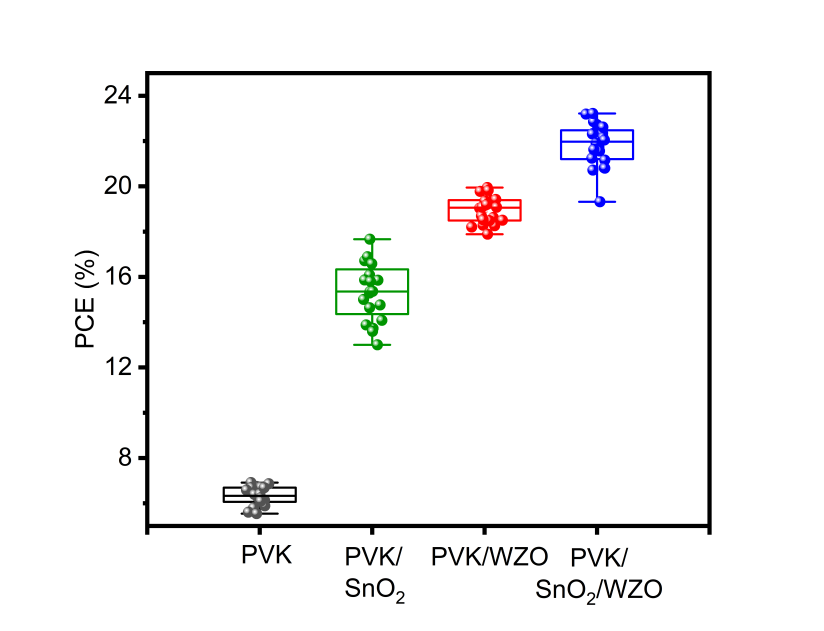


**Figure S10.** Statistical photovoltaic parameters for PVK, PVK/SnO_2_, PVK/WZO, PVK/SnO_2_/WZO devices.


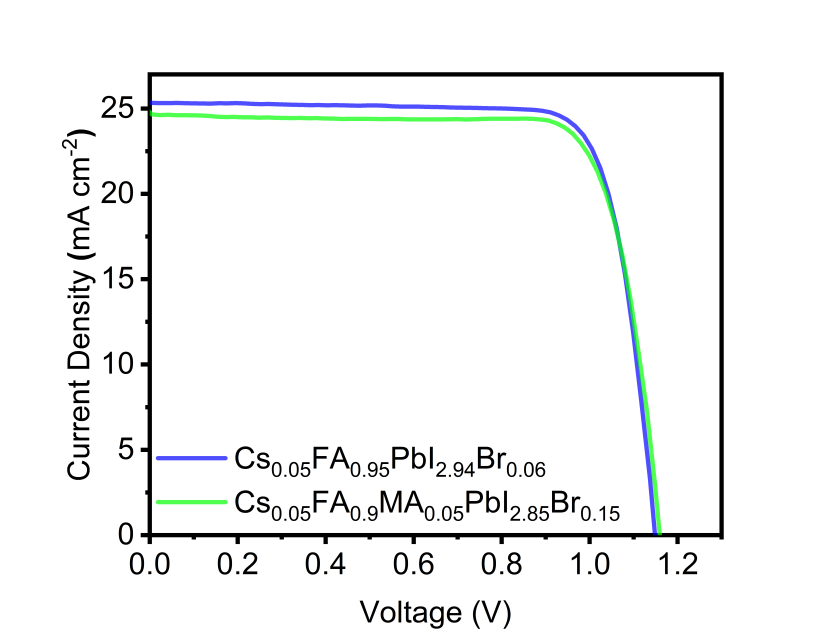


**Figure S11.** Performance for two kinds of perovskite devices.


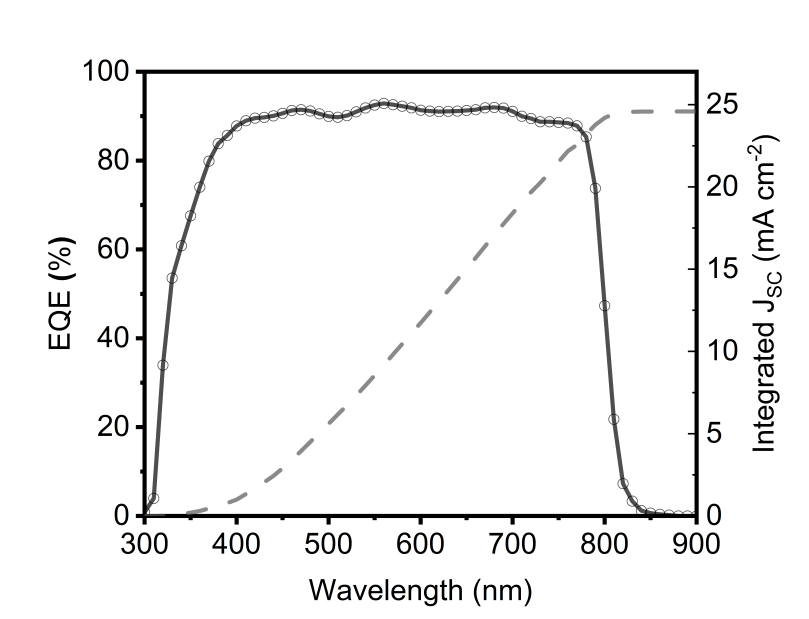


**Figure S12.** EQE curves and integrated J_SC_ for PVK/SnO_2_/WZO device.

**Table S1.** Simple resistance test by connecting different resistors in series controlled by conduction length and testing 8 nm thick SnO_2_, 8 nm thick WZO and 270 nm thick perovskite layers all with an area of 0.05 cm². The calculated average conductivity is 6.6×10^-4^ S/m for SnO_2_, 2.8×10^-3^ S/m for WZO and 1.3×10^-3^ S/m for perovskite.

| Base resistance (Ω) | 5.70 | 8.53 | 11.40 | 14.18 | 16.93 |
| --- | --- | --- | --- | --- | --- |
| Series resistor contains SnO_2_ (Ω) | 6.47 | 10.51 | 14.01 | 17.25 | 20.51 |
| Series resistor contains WZO (Ω) | 6.13 | 8.96 | 11.89 | 14.76 | 17.76 |
| Series resistor contains perovskite (Ω) | 32.90 | 43.15 | 50.99 | 57.16 | 74.68 |

**Table S2.** The key parameters for energy alignment of functional layers.

|  | CBM [eV] | VBM [eV] |
| --- | --- | --- |
| MPA-CPA | -2.6 | -5.4 |
| Perovskite | -4.1 | -5.65 |
| SnO_2_ | -4.15 | -8.17 |
| WZO | -4.29 | -7.59 |

**Table S3.** The key TRPL parameters of perovskite films by PVK, PVK/SnO_2_, PVK/WZO, PVK/SnO_2_/WZO pieces.

|  | A_1_ | t_1_ [ns] | A_2_ | t_2_ [ns] | A_3_ | t_3_ [ns] | t_average_ [ns] |
| --- | --- | --- | --- | --- | --- | --- | --- |
| PVK | 0.30006 | 4.84258 | 0.336 | 60.71496 | 0.13547 | 277.35392 | 196.32161 |
| PVK/SnO_2_ | 0.95181 | 7.85018 | 0.11058 | 53.64103 | 0.08287 | 435.50259 | 325.17667 |
| PVK/WZO | 67.06446 | 2.02392 | 66.34788 | 2.02375 | 0.25421 | 29.33969 | 2.75810 |
| PVK/SnO_2_/WZO | 0.19291 | 35.70152 | 0.77063 | 9.02012 | 0.00716 | 300.47099 | 59.72635 |

Fitting Function Formula:

y = y0 + A1*exp(-(x-x0)/t1)*(1-erf(-(x-x0-s^2/t1)/s)) + A2*exp(-(x-x0)/t2)*(1-erf(-(x-x0-s^2/t2)/s)) +A3*exp(-(x-x0)/t3)*(1-erf(-(x-x0-s^2/t3)/s))

**Table S4.** Key photovoltaic parameters for PVK, PVK/SnO_2_, PVK/WZO, PVK/SnO_2_/WZO pieces.

|  | *V_OC_*  [V] | *J_SC_*  [mA cm^-2^] | FF  [%] | PCE  [%] |
| --- | --- | --- | --- | --- |
| PVK | 0.873 | 13.99 | 54.80 | 6.69 |
| PVK/SnO_2_ | 1.038 | 23.79 | 65.13 | 16.09 |
| PVK/WZO | 1.079 | 24.99 | 72.18 | 19.47 |
| PVK/SnO_2_/WZO | 1.148 | 25.34 | 79.69 | 23.19 |

**Table S5.** Key photovoltaic parameters for concentration of spin-coated tin oxide nanoparticles by PVK/SnO_2_/WZO pieces.

|  | *V_OC_*  [V] | *J_SC_*  [mA cm^-2^] | FF  [%] | PCE  [%] |
| --- | --- | --- | --- | --- |
| w/o SnO_2_ | 1.079 | 24.99 | 72.18 | 19.47 |
| SnO_2_ 2 mg/mL | 1.124 | 25.11 | 76.96 | 21.71 |
| SnO_2_ 4 mg/mL | 1.148 | 25.35 | 79.69 | 23.19 |
| SnO_2_ 6 mg/mL | 1.161 | 25.34 | 71.44 | 21.01 |
